# Vascular insufficiency in the extremities following jellyfish-sting envenomation in Malaysia

**DOI:** 10.1016/j.toxcx.2025.100239

**Published:** 2026-01-21

**Authors:** Nur Afiqah Kamsani, Lay Tin Tan, Mohamad Muhyiddin Khalid, Ahmad Khaldun Ismail

**Affiliations:** a Department of Emergency Medicine, Faculty of Medicine, Universiti Kebangsaan Malaysia, Hospital Canselor Tuanku Muhriz, Jalan Yaacob Latif, Bandar Tun Razak, Cheras Kuala Lumpur, 56000, Malaysia; b Department of Paediatrics, Hospital Pulau Pinang, Jalan Residensi, George Town, Pulau Pinang, 10450, Malaysia

**Keywords:** Arterial vasospasm, Emergency, Iloprost, Paediatrics

## Abstract

**Introduction:**

Vascular insufficiency is an uncommon but potentially limb-threatening complication of jellyfish envenomation, with limited documentation in Southeast Asia. This study characterizes its epidemiology, clinical course, and response to targeted vasodilator therapy in Malaysia.

**Methods:**

A retrospective cohort review was conducted for confirmed jellyfish-sting cases complicated by vascular insufficiency reported to the Remote Envenomation Consultancy Services (RECS) from 2017 to 2022. Demographic, geographic, clinical, and management data were extracted from RECS consultation logs. Serial Doppler ultrasound findings and treatment responses were analysed descriptively.

**Results:**

Among 105 jellyfish sting consultations, four (3.8 %) cases of vascular insufficiency were identified, all occurring in coastal Penang and affecting children aged 7–12 years. All primary stings involved the upper limbs; two had concurrent lower limb lesions. The onset of peripheral numbness and cyanosis occurred on day three post-sting. Doppler ultrasonography revealed subcutaneous oedema, reduced arterial calibre, and diminished flow velocities consistent with arterial vasospasm. All patients received intravenous iloprost (0.5–2 ng/kg/min) with gradual tapering, guided by clinical and Doppler parameters. Rapid improvement in perfusion was documented in all cases, with minimal adverse effects (vomiting, dyspnoea, haematuria). Hospitalization lasted 12–32 days. Three patients achieved full functional recovery; one had residual scarring and contracture. No deaths occurred.

**Conclusion:**

Delayed-onset arterial vasospasm can complicate paediatric jellyfish stings, particularly in the upper limb. Early recognition of evolving ischemic signs and timely initiation of iloprost with structured tapering may avert tissue loss. Broader drug availability and standardized treatment algorithms are needed to optimize outcomes in resource-limited coastal settings.

## Highlights

• Jellyfish sting envenoming can cause delayed-onset vasospasm in children.
• Serial monitoring for pain, discoloration, swelling, and numbness is vital.
• Intravenous iloprost effectively reverses limb ischemia from jellyfish envenomation.
• Gradual iloprost tapering with Doppler Ultrasonography minimizes rebound vasospasm.

## Graphical abstract

**Figure:**
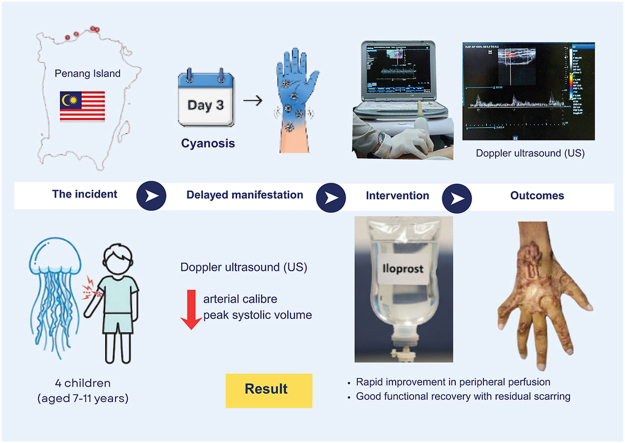

## 1 Introduction

Jellyfish stings are the most common marine injuries in Malaysia; however, most cases remain unidentified. There is inadequate documentation of harmful jellyfish species in Malaysian waters (Sivanasworn et al., 2023; Tan et al., 2022). Several jellyfish sting envenoming cases caused serious injuries and even death. A study on the west coast of Peninsular Malaysia showed that stings most often occur during clear weather, low average rainfall, and high sea temperature (Mubarak et al., 2021).

The appearance of skin lesions depends on the type of jellyfish that caused the sting. Many will have a non-specific feature, unlike the multitentacled box jellyfish species. The severity of envenomation also varies depending on the jellyfish species and the amount of venom injected. Most jellyfish stings in Malaysia cause local effects, such as pain, swelling, skin lesions, blisters, and rashes. Some may present with headache, back pain, restlessness, profuse sweating, chest tightness, abdominal discomfort, vasospasm, hypertension, muscle cramps, nausea and vomiting, myonecrosis, or cardiac arrhythmias (Mohd Suan et al., 2016; Lippmann et al., 2011; Mubarak et al., 2021; Sivanasworn et al., 2023; Tan et al., 2022). Serious complications may include secondary bacterial infections, neurological manifestations, and cardiorespiratory manifestations. There have been several deaths following multi-tentacled box jellyfish envenomation in Langkawi Island, Pangkor Island, and the coastal waters of Sabah (Lippmann et al., 2011). The characteristics or distinguishing features of jellyfish stings will depend on the type of jellyfish that a person is in contact with, which may or may not produce significant signs and symptoms of envenomation.

Vascular insufficiency is an uncommon complication of a jellyfish sting envenomation in Malaysia. However, the incidence was poorly documented in the past. Several case reports of peripheral arterial spasms following jellyfish stings have been reported in other countries (Choong et al., 2015; El Khatib and Al Basti, 2000; Lam et al., 2014; Williamson et al., 1988). The envenomation resulted in peripheral vascular spasm and digit necrosis. Surgical interventions with fasciotomy were found to be unsuccessful. However, several cases had been treated using intravascular medications (Abu-Nema et al., 1988; Binnetoglu et al., 2013; Lo et al., 2016). It appears that the vasospasm responded positively to Iloprost. There were no prior records or publications regarding the medical management of this clinical predicament in Malaysia.

Remote Envenomation Consultancy Services (RECS) is a specialized risk management support system for healthcare professionals in managing clinical toxinology-related cases. Since its inception in 2012, RECS consultation has been conducted via smartphone using text messaging platforms. Through RECS consultation, clinical data for each case are well documented, as they are typically recorded throughout a patient's management and progress until safe discharge. The RECS consultation log provides a verified source of clinical data, particularly species identification, clinical presentation, incident location, progression, management, complications, and outcomes. RECS consultation also provides a unique opportunity for direct communication among specialties managing the same case, ensuring the continuity of appropriate care and documentation. RECS offers the opportunity to minimize management errors among healthcare professionals and optimize patient care through direct communication with experts in the field. It enables active teaching and learning activities during consultations.

The objectives of this study were to analyse the frequency, geographical distribution, clinical presentations, clinical management, and treatment outcomes of vascular insufficiency following jellyfish stings referred to the RECS in Malaysia.

## 2 Methodology

This is a retrospective cohort study of confirmed jellyfish stings that cause vascular insufficiency in Malaysia, which has been consulted to RECS since 2017. Approval from the institutional research ethics committee (project code: FF-2023-361) and the RECS coordinator was obtained prior to data collection. All data were collected from the RECS consultation log and kept anonymous and confidential. The demography of each case, geographical location of the incident, clinical presentations, treatment, and outcome were documented in a standardised data collection form. Data were descriptively analysed.

## 3 Results

**Fig. 1:**
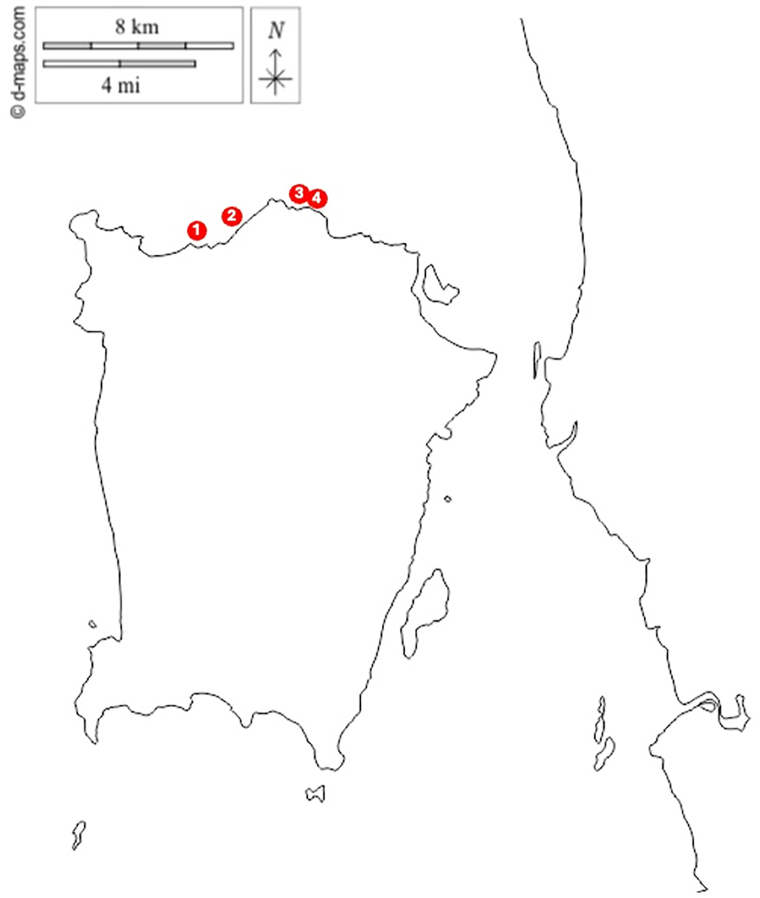
Incident locations of jellyfish cases that caused vascular insufficiency in Penang Island. Case 1 occurred at the beach of Tropical Spice Garden, Case 2 and Case 3 at Miami Beach, and Case 4 at Batu Feringghi.

A total of 105 jellyfish-related injuries nationwide were consulted to RECS within the study period. Fourteen incidents occurred in Penang, which accounts for 14.7 % of total cases. Four cases of vascular insufficiency were identified. All cases occurred in the coastal waters of Penang, Peninsular Malaysia (Fig. 1). All four cases involved children aged 7–11 years (Table 1).

Base map static image from https://d-maps.com/m/asia/malaisie/penang/penang32.gifNote: map lines delineate study areas and do not necessarily depict accepted national boundaries

**Table 1:** Details summary of clinical manifestation, treatment, and outcome of the four victims of vascular insufficiency following unidentified jellyfish stings.

Cases	1	2	3	4
Date and time of incident	February 2017 1500H	November 2020 time unspecified	November 2020 time unspecified	December 2022 1500H
Time first presented to clinic/GP	unspecified	within 2 h post-stung	within 2 h post-stung	unspecified
Day admitted to the hospital post-incident	Day 3	Day 8	Day 9	Day 2
Gender	Male	Male	Female	Male
Age (years old)	11	9	7	11
Anatomical distribution	arm, forearm, hand, thigh	arm, forearm	arm, forearm	forearm, hand, foot
First aid administered	vinegar	vinegar	vinegar	water
Chief complaint	blurring of vision, fitting episode	cyanosis of the affected hand	extreme pain and unable to move the affected hand	cyanosis of the affected hand
Onset of presentation				
Local				
Pain	Immediate	Immediate	Day 6	Day 3
Swelling	Immediate	Immediate	Day 5	Day 3
Peripheral numbness	Day 1	Unspecified	Unspecified	Day 3
Peripheral cyanosis	Day 6	Day 6	Day 10	Day 3
Blister	No	No	No	Day 11
Dermonecrosis	No	No	No	Day 11
Systemic				
Fever	Day 1	No systemic manifestation	No systemic manifestation	No systemic manifestation
Headache	Day 4			
Nausea/vomiting	No			
Visual impairment	Day 4			
Convulsion	Day 4			
Breathlessness	Day 4			
Hypertension	Day 4			
Altered mental status	Day 4			
Initial vital signs				
Blood pressure (mmHg)	160/90	110/69	100/62	116/69
Pulse rate (bpm)	110	80	100	95
Saturation oxygen (%)	99	99	98	Unspecified
Respiratory rate (bpm)	24	20	22	22
Treatment				
Antibiotics	Yes	No	Yes	Yes
Analgesia	Yes	Yes	Yes	Yes
Antiplatelet (Aspirin)	Yes	Yes	Yes	Yes
Iloprost	Yes	Yes	Yes	Yes
Antihypertensive	Yes	No	No	No
Length of Hospital Stay	21 days	13 days	12 days	32 days
Outcome (Morbidity)				
Scarring	No	Yes	Yes	Yes
Contracture	No	No	No	Yes
Finger shortening	No	No	No	Yes
Functionally impaired	No	No	No	Yes

All stings occurred on the upper limbs, with two cases having concurrent stings on the lower limbs. Vascular insufficiency only occurred on the upper limbs. All patients initially presented with local pain and swelling in the affected limbs. Peripheral numbness and cyanosis first appeared after day three of the incident. One child developed neurological signs and symptoms that were presented on day four post-incident with headache, nausea, vomiting, and convulsion, which required intubation. Initial Doppler ultrasound of the affected limb in all cases revealed subcutaneous oedema, reduced blood flow velocity, and reduced arterial calibre, indicating arterial vasospasm.

Intravenous iloprost infusion was initiated in all cases with a dose ranging from 0.5 ng/kg/min to 2 ng/kg/min over several days (Table 2). Repeated Doppler ultrasound is done once or twice daily to review the response. All patients responded positively, with few adverse effects, including vomiting and shortness of breath. The hospitalization lasted 12 to 32 days, and patients were eventually discharged home well. However, vascular insufficiency induced by jellyfish has resulted in morbidity such as long-term scarring, finger shortening, and functional impairment. No mortality was documented in this study.

**Table 2:** Iloprost administration and response.

Case	1	2	3	4
Iloprost administration after the incident	Day 5	Day 9	Day 10	Day 4
Maximum dosage	1.5 ng/kg/min	1.5 ng/kg/min	1.5 ng/kg/min	2 ng/kg/min
Duration of administration	16 days	10 days	10 days	16 days
Positive response	Yes	Yes	Yes	Yes
Velocity (cm/s)				
Radial	11.1 to 30.0	10.1 to 24.8	9.8 to 35.2	undocumented
Ulnar	14.2 to 34.9	10.0 to 23.8	9.6 to 28.1	undocumented
Brachial	32.2 to 47.0	18.0 to 45.6	17.5 to 43.1	undocumented
Calibrity (mm)				
Radial	small to 2.0	1.0 to 1.9	1.1 to 1.9	0.19 to 2.3
Ulnar	small to 2.0	1.5 to 2.0	1.2 to 1.8	1.6 to 2.2
Brachial	2.6 to 4.0	1.5 to 3.0	1.8 to 2.9	undocumented
Adverse effect	Vomiting	Shortness of breath	Nil	Nil

## 4 Case 1

**Fig. 2:**
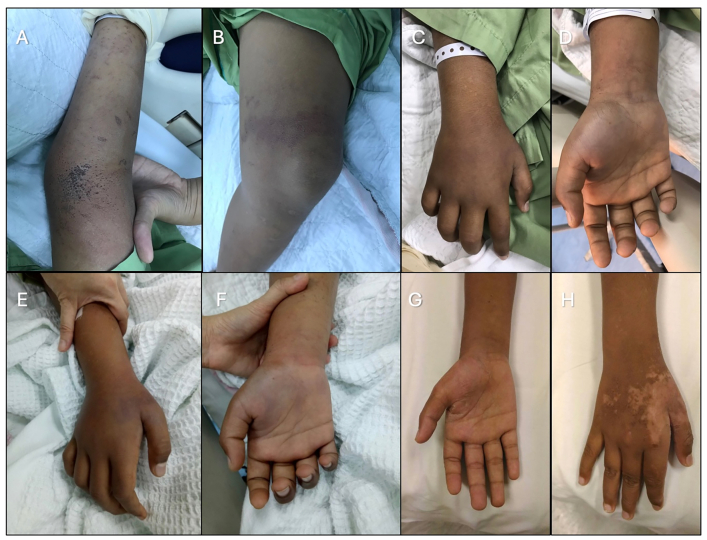
(A, B) Initial presentation on Day 4 post-envenomation showing swelling and erythema over the right arm and right thigh. Gradual improvement in discoloration and reduction in swelling was observed by Day 5 (C, D) and Day 6 (E, F), respectively, following the initiation of intravenous iloprost. Cyanosis and swelling fully resolved, with residual scarring visible over the radial side of the right hand (G, H) during follow-up on Day 35 post-incident.

A previously well 11-year-old boy sustained an unidentified jellyfish sting over his right forearm and left inner thigh while swimming at the beach in Teluk Bahang, Penang. Vinegar was applied, and he was treated conservatively with analgesia. Despite that, he still had persistent pain and swelling with right thumb numbness (Fig. 2). Four days later, he became breathless. He complained of a headache followed by visual impairment and generalized convulsions at home. He was brought to the emergency department and was intubated for airway and cerebral protection. He was found to be encephalopathic and in hypertensive crisis, possibly a delayed manifestation of systemic vasospasm. Urgent computed tomography (CT) brain revealed mild cerebral oedema with possible posterior reversible encephalopathy syndrome (PRES) changes. His blood pressure was well controlled on two anti-hypertensive medications, and he was successfully extubated after two days, with no neurological sequelae.

While being monitored closely in intensive care unit (ICU), there was right radial artery compromise distal to the wrist, evidenced by weak radial pulse and dusky right hand thenar eminence and anatomical snuff box (Fig. 2). Oxygen saturation over all fingers were undetectable and doppler ultrasound scan showing reduced blood flow for distal radial and ulnar arteries (S1 Table).

S1 Table: Case 1 serial Doppler Ultrasound of the arteries of the affected upper limb. Arteries name abbreviations: Right brachial artery (RBA), Left brachial artery (LBA), Right radial artery (RRA), Left radial artery (LRA), Right ulnar artery (RUA), Left ulnar artery (LUA).

As no specific antivenom was available, only supportive treatment with oral antibiotics and Aspirin was initially given. RECS was consulted and intravenous iloprost was initiated with dilution of 20 mcg in 100 ml normal saline (NS) at rate of 5 ml/h (0.5 ng/kg/min) over 3 h, then increased to 10 ml/h (1 ng/kg/min) over 3 h, and finally 15 ml/h (1.5 ng/kg/min) for total of 7 h (Table 3). There was no significant improvement.

**Table 3:** Case 1 Dose of iloprost administration of the affected limb based on the clinical response.

Day of incident	Infusion	Dose	Remarks
Day 5	Day 1	0.5–1.5 ng/kg/min Dilution of 20 mcg in 100 ml normal saline	
Day 6	Day 2	0.5–1.5 ng/kg/min Dilution of 50 mcg in 50 ml normal saline	Develop vomiting at a rate of 1.5 mg/kg/min
Day 7–8	Day 3–4	0.5–1.5 ng/kg/min Dilution of 50 mcg in 50 ml normal saline	
Day 9 (am)	Day 5		Withheld iloprost
Day 9 (pm)	Day 5	1.5 ng/kg/min Dilution of 50 mcg in 50 ml normal saline	Iloprost was restarted due to numbness and sensation over the right thumb and index finger and worsening discoloration.
Day10–12	Day 6–8	1.5 ng/kg/min Dilution of 50 mcg in 50 ml normal saline	
Day13–14	–		Withheld for 42 h
Day 16	Day 9	1.5 ng/kg/min	Restarted iloprost infusion due to a weaker right radial pulse compared to the left radial pulse
Day 17	Day 10	1 ng/kg/min	Rapid and sustained improvement. Tapered down the iloprost dose
Day 18	Day 11	0.5 ng/kg/min	Rapid and sustained improvement. Tapered down iloprost
Day19–22	Day 12	0.5 ng/kg/min Dilution 12.5mcg in 25 ml normal saline	
Day 23	Day 13	0.25 ng/kg/min	
Day 24			Off iloprost

The following day, a more concentrated dilution of 50 mcg in 50 ml NS was infused at a rate of 1 ml/h (0.5 ng/kg/min) for 30 min, then 2 ml/h (1 ng/kg/min) for 30 min, and finally increased to 3 ml/h (1.5 ng/kg/min) for total of 17 h. There was some clinical improvement evidenced by reduced swelling, numbness, and dusky discolouration over his right forearm and hand. By day 5 of iloprost infusion, the calibre and peak systolic velocity (PSV) of his right radial artery had normalised. The infusion was discontinued after 7 days because there were no significant changes in clinical or sonographic assessments.

Two days after discontinuation of the iloprost infusion, the boy developed sudden worsening pain, numbness, and a weaker right radial pulse with a sudden drop of PSV over the right radial artery. Iloprost was subsequently restarted at 1.5 ng/kg/min. Iloprost was tapered gradually over 1 week: 1.5 ng/kg/min for 1 day, 1 ng/kg/min for 1 day, 0.5 ng/kg/min for 5 days, and 0.25 ng/kg/min for 1 day.

After 3 weeks of admission, he was discharged home well. Prior to discharge, his anti-hypertensive medications were successfully weaned off, and he regained full function of his right upper limb. All other investigations, including echocardiogram, renal function tests, antineutrophil cytoplasmic antibody (ANCA) assays, rheumatoid factor (RF), C3 and C4 levels, cortisol, and urine biochemistry, were within the reference range, ruling out other secondary causes of hypertensive crisis and vascular insufficiency.

## 5 Case 2

A previously well 9-year-old boy was swimming in the sea of Batu Feringghi, Penang, Malaysia, with his 7-year-old sister in November 2020. The boy was stung on the left forearm; however, the jellyfish species was not identified. Post-stung, vinegar spray was applied immediately to the affected site. He was brought to a general practitioner clinic on the same day and was discharged with oral antihistamine and Paracetamol.

**Fig. 3:**
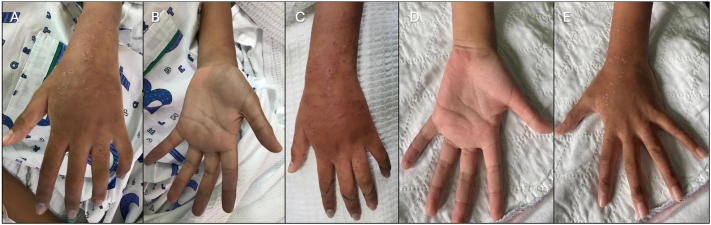
(A, B) Initial presentation on Day 8 post-envenomation revealed cyanosis affecting the tips of the left index, middle, and ring fingers, indicative of digital ischemia. (C) Perfusion improved following initiation of intravenous iloprost. (D, E) Complete resolution of cyanosis was achieved by Day 17, following 10 days of continuous iloprost infusion.

Six days later, he developed progressive swelling, tingling, and pain, followed by numbness and bluish discoloration over the tips of the left index, middle, and ring fingers. Parents brought him to the emergency department the next day. At presentation, his blood pressure was 110/69, pulse rate was 80, and SpO2 was 98 %, with no cardiorespiratory compromise. The boy had erythematous maculo-papular rash over the anterolateral aspect of the left forearm and dorsum of the left hand. The tips of his left index, middle, and ring fingers were cyanosed, cold, and tender (Fig. 3).

The left brachial pulse was strong, while the radial and ulnar pulses were palpable but weak. Capillary refill was prolonged over the left index, middle, ring, and little fingers, with oxygen saturation ranging from 85 to 88 % over the fingers. He could actively flex and extend all his fingers; however, he was unable to make a complete fist. The range of motion over the left shoulder, elbow, and wrist joints was unaffected.

RECS was consulted, and intravenous iloprost was infused on the second day of admission. Fifty micrograms of Iloprost (50 mcg/0.5 ml) was diluted in 50 ml of NS and infused at 0.1 ng/kg/min (Table 4). Vital signs were documented every 30 min after initiation of iloprost. The iloprost infusion rate was increased progressively until the target rate of 1.5 ng/kg/min was achieved without adverse effects. The iloprost infusion was run hourly for 24 h. He was also started on oral Aspirin (5 mg/kg/day) and intravenous Augmentin for a marine sting. Other supportive management included pain control, limb elevation, and slit lamp treatment. Doppler ultrasound was performed twice daily to assess the objective response to therapy.

**Table 4:**
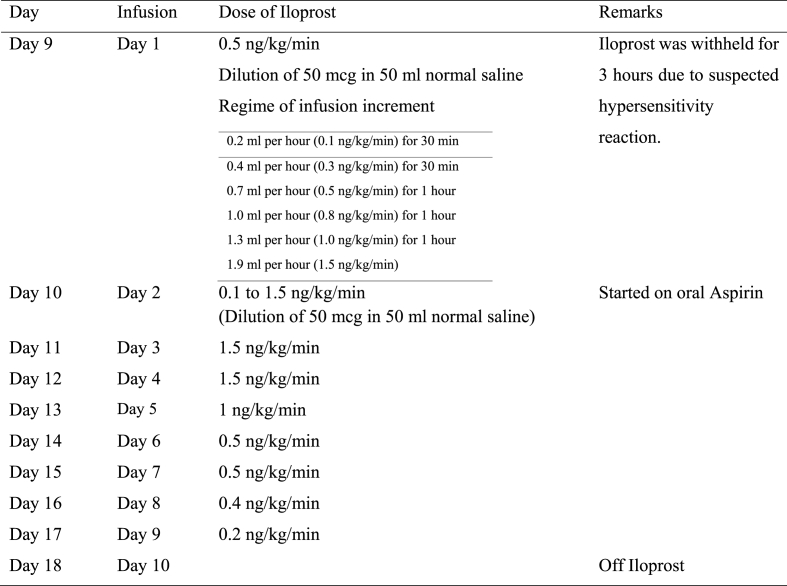
Case 2 Dose of iloprost administration of the affected limb based on the clinical response.

There was a gradual, marked clinical improvement. He tolerated the infusion well, with no headache, gastrointestinal symptoms, namely abdominal cramps, nausea and vomiting, hypotension or change in heart rate. Perfusion of the affected fingers was notably improved, with radial and ulnar pulses progressively stronger after day 2 of iloprost infusion. The tenderness of the digits resolved after 5 days of iloprost infusion. The calibres and PSV also improved at the end of the treatment, as tabulated above. The iloprost infusion was tapered to 1.0 ng/kg/min on day five and successfully discontinued after ten days of treatment. During the first tapering period, the orthopaedics team applied topical Glyceryl trinitrate (GTN) patches without significant changes, which were subsequently discontinued.

He was monitored for 1 day after Iloprost was discontinued and was discharged with minimal local symptoms in the affected limbs. He was discharged with oral Aspirin and analgesia. During the outpatient clinic follow-up one week after discharge, his affected upper limbs had regained full function with normal Doppler ultrasound findings.

## 6 Case 3

A previously well 7-year-old girl was swimming in the sea at Batu Ferringhi, Penang, Malaysia, with her 9-year-old brother. She was stung by jellyfish on her right-sided back and her right upper limb. The girl developed swelling over the right upper limb, with sudden onset of erythema; subsequently, worsening pain along the right forearm to her wrist and dorsal aspect of her right hand, followed by extreme pain, numbness, and gradual loss of motion of her right upper limb over the next seven days.

**Fig. 4:**
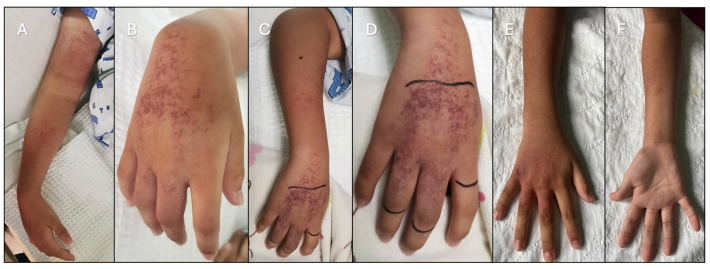
(A, B) Initial presentation showing a diffuse erythematous vesiculopapular rash over the dorsum of the right hand with pallor at the fingertips. Findings consistent with early inflammatory and ischemic changes following jellyfish envenomation. (C, D) Clinical improvement was observed following initiation of intravenous iloprost, with a reduction in swelling and rash. (E, F) At the time of discharge, swelling had resolved, with residual cutaneous erythema and minimal vesicular changes remaining on the affected upper limb.

There was a well-demarcated purpuric rash with swelling extending from the right arm to the hand (Fig. 4). The muscular compartments of her arm and forearm were tense. The tips of her right index, middle, ring, and little fingers were pale, cold, and tender. There was minimal range of motion: she was unable to fully abduct, adduct, flex, extend, or rotate at her shoulder joint, and to flex or extend at her elbow joint. She was only able to flex and extend very minimally at all distal interphalangeal joints. The right brachial, radial, and ulnar pulses were not palpable. Capillary refill was prolonged for all fingers. Saturation ranged 80 %–85 % over the right index, middle, and ring fingers, while the right thumb and little finger had documented saturation of 95 %–96 %. Initial ultrasound Doppler of the right upper limb showed good colour flow and normal Doppler waveform over the right brachial, ulnar, and radial arteries. Urgent computed tomography angiography (CTA) of the right upper limb demonstrated poor contrast opacification beyond the right brachial artery and was unable to exclude thrombosis. She was referred to the orthopaedic team, with compartment syndrome ruled out.

RECS was consulted and advised that a jellyfish sting could lead to vascular insufficiency and severe digital ischemia. She was started on intravenous Iloprost infusion on the following day, due to the onset of a tinge of cyanosis over the tips of the fingers, more notably over the right index, middle, and ring fingers after admission (Table 5). Fifty micrograms of iloprost (50 mcg/0.5 ml) was diluted into 50 ml of NS. Every 30 min after initiation of the iloprost infusion and increment, the vital signs were checked and monitored.

**Table 5:**
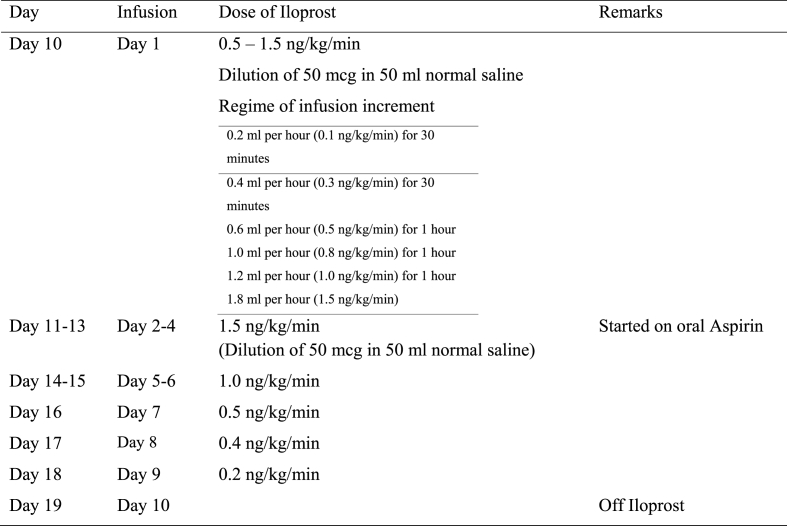
Case 3 Dose of iloprost administration of the affected limb based on the clinical response.

The iloprost infusion was run hourly for 24 h. She was started on oral Aspirin (5 mg/kg/day) and intravenous Augmentin for a marine sting. Other supportive management included pain control, limb elevation, and slit lamp treatment. Doppler ultrasound was performed twice daily to assess the objective response to therapy.

There was a gradual, marked clinical improvement in the affected limb. She tolerated the infusion well, with no headache, gastrointestinal symptoms, namely abdominal cramps, nausea and vomiting, hypotension or change in heart rate. Perfusion of the affected fingers was notably improved, with radial and ulnar pulses progressively stronger after day 2 of Iloprost infusion. The tenderness of the digits resolved after 5 days of iloprost infusion. The calibres and PSV also improved at the end of the treatment, as tabulated above.

The iloprost infusion was tapered to 1.0 ng/kg/min on day five of treatment and was gradually reduced, and successfully discontinued after ten days. During the first tapering period, topical GTN patches were used. She was monitored for a day and allowed discharge with minimal local symptoms over the affected limbs, and was discharged with oral Aspirin and analgesia. At the follow-up clinic one week after discharge, the affected upper limbs had regained full function, with normal Doppler ultrasound findings.

## 7 Case 4

An 11-year-old boy sustained an unidentified jellyfish sting on his right hand and both feet at Batu Feringghi, Penang, in December 2022. Drinking water was applied, and he was treated as an outpatient with intravenous Hydrocortisone, Chlorphenamine, and Paracetamol. Three days later, his swelling, pain, and numbness over his right hand worsened and intensified. The initial Doppler ultrasound showed a monophasic waveform over the radial and distal ulnar arteries; a biphasic waveform over the proximal ulnar artery (S2 Table).

S2 Table: Case 4 serial Doppler ultrasound findings. Arteries name abbreviations: Right brachial artery (RBA), Right radial artery (RRA), Right ulnar artery (RUA), Dorsalis pedis artery (DPA), posterior tibial artery (PTA)

**Fig. 5:**
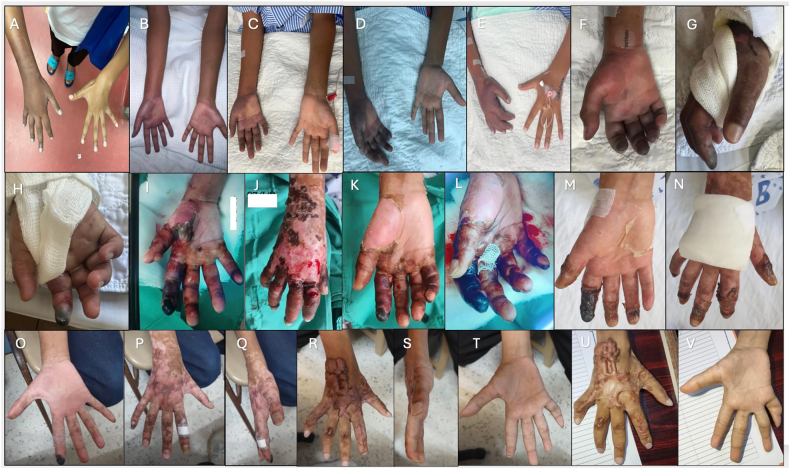
(A, B) Initial presentation showing dusky discoloration of the right hand with swelling extending to the mid-forearm, consistent with early signs of vascular compromise. (C–E) Progressive clinical improvement following intravenous iloprost infusion, with resolution of cyanosis and reduction in swelling over the course of 1, 2, and 4 days, respectively. (F–H) However, due to an unexpected interruption in iloprost supply, the infusion was discontinued abruptly, resulting in clinical deterioration (I–L). Over the following three days, the patient developed worsening oedema and blister formation on the right palm, dorsum, and wrist, as well as dusky discoloration of the right index finger; therefore, iloprost was restarted. The patient was discharged after completing 19 days of iloprost therapy, with a significant reduction in swelling and residual necrotic changes at the fingertip (M, N). At three weeks post-discharge, follow-up images demonstrated marked improvement with healing and scarring (O–Q). At two years post-envenomation, the patient exhibited fibrotic scarring on the affected limb, with no evidence of recurrent ischemia (R–V).

The following day, the right-hand swelling extended up to his mid-forearm. Clinically, there were weak radial and ulnar pulses, dusky discolouration over his right hand, and undetectable saturation over all fingers of his right hand (Fig. 5). Doppler ultrasound demonstrated absent blood flow for the distal part of the radial and ulnar arteries. CTA angiogram was done, confirming vasospasm of the right radial and ulnar arteries. Iloprost infusion was initiated on day 4 of the incident at 0.8 ml/h (0.5 ng/kg/min) for 3 h, then increased to 1 ng/kg/min for 3 h, and finally to 2.0 ng/kg/min (Table 6). The infusion was run for 24 h daily. Additionally, supportive treatment was provided, including a warming blanket, a right-hand resting splint, and analgesia (Paracetamol). Oral Tab Cardiprin 100 mg daily and intravenous Heparin were also started, alongside GTN patches.

**Table 6:** Case 4 Dose of iloprost administration of the affected limb based on the clinical response.

Day of incident	Infusion	Dose of iloprost	Remarks
Day 4	Day 1	0.5–2 ng/kg/min titrated slowly Dilution 50mcg in 50 ml normal saline	
Day 5	Day 2	2 ng/kg/min	Improvement of cyanosis
Day 6	Day 3	2 ng/kg/min (for 16 h only)	Stop due to the unavailability of iloprost
Day 7–9	Day 4–6	2 ng/kg/min	Iloprost was stopped on day 6 of infusion
Day 12	Day 7–10	2 ng/kg/min	Restarted iloprost in view of persistent blackish discoloration of the right index finger and swelling of the right hand
Day 16	Day 11		Withheld due to haematochezia and haematuria
Day 18	Day 12	1.5 ng/kg/min	Restarted iloprost
Day 19	Day 13	1 ng/kg/min	
Day 20–25		0.5 ng/kg/min for 5 days.	Tapered down and off
Day 32			Discharge

There was clinical improvement after six days of iloprost infusion. The Doppler monitoring showed a triphasic waveform of the right radial artery, with normalised calibre of the affected artery. Due to the unavailability of iloprost, the infusion was abruptly discontinued. Oral Aspirin and GTN patches were continued. However, there was progressive deterioration over the next three days, with worsening swelling and blister formation on his right palm, dorsum, and wrist, and dusky discoloration of his right index finger.

Iloprost infusion was restarted at 2 ng/kg/min, administered continuously for 24 h daily. Hand circulation gradually improved over the next few hours. After three days of infusion, he developed an episode of haematochezia and haematuria. The infusion was withheld for two days before restarting. During these two days, there was no worsening of right-hand circulation; thus, Iloprost was gradually weaned over 1 week: 1.5 ng/kg/min for 1 day, 1 ng/kg/min for 1 day, and 0.5 ng/kg/min for 5 days. The boy was discharged with a residual necrotic patch at the tip of his right index finger. All other investigations had ruled out possible secondary causes.

## 8 Discussion

Limb vascular insufficiency secondary to jellyfish envenomation is a rare but may result in a permanent disabling complication if not promptly recognized and treated. In our study, all four patients presented with progressively worsening localized pain and swelling of the affected area, despite receiving analgesia and supportive care at home. Delayed neurological and vascular manifestations, including peripheral numbness and cyanosis, became apparent by day four post-envenomation. Notably, only Case 1 developed systemic envenomation, characterized by nausea, vomiting, and seizures, requiring endotracheal intubation. Although two patients sustained additional stings to the lower limbs, vascular insufficiency was observed exclusively in the upper limbs affected in the initial stings.

To date, there are no established clinical guidelines for the management of vascular insufficiency following jellyfish envenomation (Lam et al., 2014; Lo et al., 2016). However, case reports by (Badran et al., 2022; Binnetoglu et al., 2013; Lo et al., 2016) have demonstrated successful outcomes using intravenous iloprost infusion in managing ischemic complications without the need for surgical intervention. In these cases, patients presented with early red-flag symptoms suggestive of arterial insufficiency and vasospasm, including numbness, progressive swelling, worsening pain, and bluish discoloration. Doppler ultrasound of the affected limb demonstrated monophasic flow, suggestive of peripheral digital vasospasm. Conventional vasodilators such as nitrates and calcium channel blockers were ineffective as first-line treatment (Badran et al., 2022; Binnetoglu et al., 2013; Lo et al., 2016). In contrast, the administration of iloprost not only halted the progression of ischemia but also significantly reduced the risk of surgical intervention and shortened the duration of hospitalization (Badran et al., 2022; Lo et al., 2016).

Iloprost, a synthetic analogue of prostacyclin (PGI_2_), is a potent but short-acting vasodilator with a half-life of approximately 20–30 min. In addition to inducing vasodilation, iloprost also exerts antithrombotic effects by inhibiting platelet aggregation, making it a valuable agent in the management of vascular insufficiency and ischemic conditions (Lo et al., 2016). Iloprost is recognized for managing acute vasospastic crises, notably in scleroderma, where it provides acute stabilization before being tapered following the introduction of definitive treatments like corticosteroids and maintenance therapy (Binnetoglu et al., 2013). Standard guidelines generally recommend infusion rates up to 2 ng/kg/min (Badran et al., 2022; Ingegnoli et al., 2019) although expert consensus suggests doses as high as 10 ng/kg/min may be warranted for severe vasospasm (Shann, 2017)

**Fig. 6:**
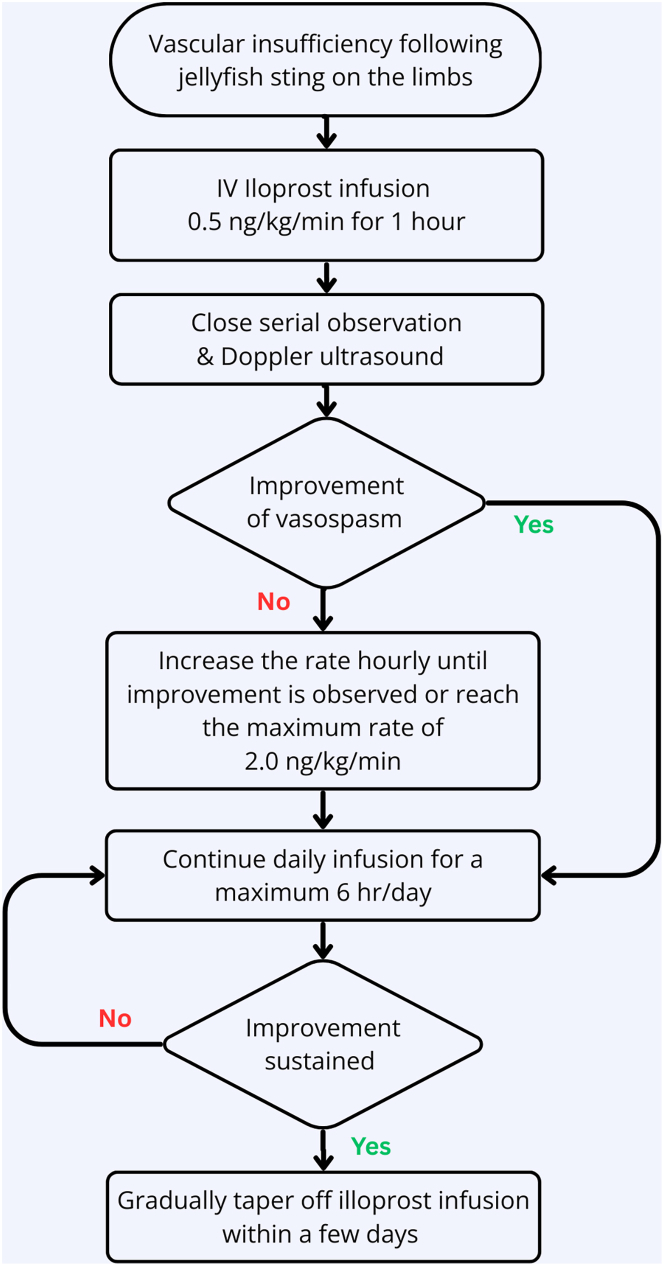
Recommended clinical management flow chart for vascular insufficiency following jellyfish sting envenoming.

Iloprost infusion was administered at doses up to 2 ng/kg/min in the four reported cases of jellyfish-sting-induced severe vasospasm. Serial Doppler ultrasound assessments were performed once or twice daily, demonstrating progressive improvement in arterial calibres and PSV over several days. Minimal adverse effects, including vomiting, shortness of breath, and haematuria, were recorded; however, no severe adverse events were observed at this dosage. Here, we include the management flow chart that may assist clinicians in managing such cases in the future (Fig. 6)

In both Case 1 and Case 4, premature discontinuation of iloprost infusion was associated with recurrence and worsening of local circulatory compromise. In Case 1, the patient experienced increased pain, numbness, weakened right radial pulse, and a sudden drop in PSV on Doppler assessment following early cessation of the infusion. Upon reinitiation, iloprost was administered and gradually tapered over the course of a week, resulting in clinical improvement and stabilization of vascular parameters. These suggest that a gradual tapering of iloprost infusion is advisable to prevent rebound ischemic symptoms and ensure sustained vascular recovery.

The decision to initiate tapering down the iloprost infusion was guided by (1) clinical judgment of the improving peripheral perfusion and stronger distal pulses, and (2) improvement in the calibres and PSV, as well as the serial ultrasound. Iloprost can be further reduced if there is no deterioration in clinical signs or ultrasound findings during tapering for a total of 1 week. Based on serial clinical observations, the application of a GTN patch did not produce a significant clinical improvement in peripheral vasoconstriction following jellyfish envenomation. In cases where iloprost infusion was discontinued, the use of a GTN patch on the affected limb neither worsened nor improved the vascular status (Badran et al., 2022).

The precise pathophysiological mechanism of jellyfish venom-induced localized vascular compromise remains uncertain. However, according to Williamson et al., many jellyfish toxins induce vasoconstriction, elevate systemic blood pressure, and may influence the autonomic nervous system. Therefore, it is plausible that the reduction in peripheral blood flow is attributed to a combination of direct vascular effects of the venom, neurogenic mechanisms, reflex sympathetic activation, or an interplay of all three (Williamson et al., 1988).

GTN primarily acts on the systemic venous system and is a more potent venodilator than an arterial dilator. It exerts its pharmacologic effect by releasing nitric oxide (NO) (Pearson and Butler, 2021), which activates guanylate cyclase, thereby increasing intracellular cyclic guanosine monophosphate (cGMP) levels. Elevated cGMP levels promote dephosphorylation of myosin light chains, resulting in vascular smooth muscle relaxation and subsequent vasodilation (Kim et al., 2025) This vasodilation causes peripheral pooling of blood, thereby decreasing preload and reducing both ventricular volume and intracardiac pressures. Consequently, myocardial wall tension and oxygen demand are lowered. However, GTN does not significantly increase cardiac output (Baysal et al., 2006) and is, therefore, limited in its ability to reverse high-resistance arterial insufficiency.

In contrast, the primary mechanism of iloprost involves activation of the prostacyclin (IP) receptor (Lai et al., 2012). This stimulates adenylate cyclase, increasing intracellular cyclic adenosine monophosphate (cAMP) levels (Krug et al., 2009) which in turn promotes relaxation of vascular smooth muscle. Iloprost induces vasodilation of both systemic and pulmonary arteries, leading to a reduction in systemic and pulmonary vascular resistance, which reduces afterload and consequently increases stroke volume and cardiac output (Baysal et al., 2006; Joshi et al., 2020). This enhanced perfusion through previously constricted arterial beds results in a consistent and significant improvement in hemodynamics. Given its broader and more effective vasodilatory effect, iloprost demonstrates superior therapeutic potential compared to GTN in managing vascular insufficiency associated with jellyfish envenomation.

While clinical management of jellyfish envenomation is essential, initial first-aid measures also play a critical role in preventing the severity of local and systemic complications. Current first-aid strategies aim to (1) alleviate the local effects of venom, (2) prevent further discharge from nematocysts, and (3) manage systemic reactions, including anaphylaxis and shock (Cegolon et al., 2013). Immediate retrieval of the victim from the water and initiation of appropriate life support measures are the priorities. The early application of vinegar has been traditionally used to inhibit further nematocyst discharge; however, this method has recently controversial (Welfare et al., 2014). In this case series, three patients received immediate vinegar application following the sting, before the vasospasm became apparent several days later.

## 9 Limitations

Several limitations of this case report warrant consideration. The findings may not be generalisable due to geographical and demographic constraints, as patients are limited to RECS consultation, which may not reflect the exact total number of jellyfish envenomations in Malaysia. Additionally, identification of the jellyfish species responsible for envenomation was not achieved in all cases, limiting the ability to correlate specific venom profiles with clinical manifestations and treatment outcomes. Additionally, limitations in the measurement and interpretation of Doppler ultrasound for vascular assessment are influenced by operator skill, technique, and interobserver variability. The lack of iloprost weaning and hemodynamic monitoring protocols, along with the absence of established reference ranges for Doppler ultrasound measurements specific to the pediatric population, also complicates the interpretation and assessment of vascular findings. A trend-based evaluation using serial Doppler ultrasound to monitor temporal changes is recommended. Additionally, the use of iloprost is limited by its availability in some government hospitals in Malaysia due to cost constraints and its infrequent use in clinical practice, which may affect treatment options. Therefore, further dedicated research is necessary to determine the optimal, cost-effective dosing regimen for iloprost and to establish evidence-based guidelines for its use in this specific clinical context. It is recommended that patients presenting with red flag symptoms for vascular insufficiency, such as persistent pain, swelling, and numbness, particularly in the upper limb, should undergo close clinical monitoring, as delayed manifestations often emerge between the third and fifth day post-incident.

## 10 Conclusion

Iloprost has demonstrated a positive vasodilatory effect in our patients, with evidence of progressive return of radial and ulnar pulses during iloprost infusion. Despite this, the current literature lacks a clear consensus on the optimal management of jellyfish-sting-induced vascular insufficiency, with no standardized or algorithmic approach having yet been established. The development of evidence-based protocols and improved access to Iloprost in resource-limited settings will be critical to improving care for envenomated patients. Prompt recognition of red flag symptoms, particularly in upper-limb involvement, and early initiation of iloprost infusion with gradual tapering based on clinical and Doppler trends are essential to prevent progression of vascular insufficiency. Clinicians should maintain a high index of suspicion for delayed vascular manifestations following jellyfish stings, especially when symptoms such as pain, numbness, or cyanosis emerge several days post-exposure.

## CRediT authorship contribution statement

**Nur Afiqah Kamsani:** Writing – original draft, Methodology, Formal analysis, Data curation. **Lay Tin Tan:** Writing – original draft, Methodology, Formal analysis, Data curation. **Mohamad Muhyiddin Khalid:** Writing – original draft, Data curation. **Ahmad Khaldun Ismail:** Writing – original draft, Validation, Supervision, Project administration, Methodology, Formal analysis, Data curation, Conceptualization.

## Ethical statement

Hereby, I, Ahmad Khaldun Ismail, consciously assure that the manuscript “Vascular insufficiency in the extremities following jellyfish-sting envenomation in Malaysia”, the following is fulfilled.

1) This material is the author's own original work, which has not been previously published elsewhere.
2) The manuscript is not currently being considered for publication elsewhere.
3) The manuscript reflects the authors' own opinion in a truthful and complete manner.
4) The manuscript properly credits the meaningful contributions of co-authors.
5) All sources used are properly disclosed.
6) All authors have been personally and actively involved in drafting this correspondence manuscript and will take public responsibility for its content.

I agree with the above statements and declare that this submission follows the policies of Toxicon X as outlined in the Guide for Authors and in the Ethical Statement.

## Declaration of generative AI and AI-assisted technologies in the writing process

During the preparation of this work, the author used Grammarly to guide the writing process. After using this service, the author reviewed and edited the content as needed and takes full responsibility for the published article.

## Declaration of competing interest

The authors declare that they have no known competing financial interests or personal relationships that could have appeared to influence the work reported in this paper.

## Data Availability

No data was used for the research described in the article.
