# Supplementary material for: Vascular insufficiency in the extremities following jellyfish-sting envenomation in Malaysia
Source: Toxicon X. 2026 Jan 21;29:100239. doi: 10.1016/j.toxcx.2025.100239 (PMC12870873; doi:10.1016/j.toxcx.2025.100239)
Supplement: Multimedia component 1 [file mmc1.docx]

S1 Table: Case 1 serial Doppler Ultrasound of arteries of affected upper limb. Arteries name abbreviations: Right brachial artery (RBA), Left brachial artery (LBA), Right radial artery (RRA), Left radial artery (LRA), Right ulnar artery (RUA), Left ulnar artery (LUA).

| Day of incident | Caliber (mm) | | | | | | PSV (cm/sec) | | | | | | Color Doppler | | | | | |
| --- | --- | --- | --- | --- | --- | --- | --- | --- | --- | --- | --- | --- | --- | --- | --- | --- | --- | --- |
|  | RBA | LBA | RRA | LRA | RUA | LUA | RBA | LBA | RRA | LRA | RUA | LUA | RBA | LBA | RRA | LRA | RUA | LUA |
| Day 5 |  |  |  |  |  |  | 32.2 | 33.2 | 11.1 |  | 14.2 | 27.9 | Moderate | Good | Weak | Good | Weak | Good |
| Day 6 |  |  | Small |  | Small |  |  |  |  |  |  |  | Normal | Normal | Weak | Good | Weak | Good |
| Day 7 |  |  | Small |  | Small |  |  |  |  |  |  |  | Normal |  | Present |  | Present |  |
| Day 8 | 2.6 |  | 1.2 – 2.0 |  | 1.4 - 3 |  |  |  |  |  |  |  | Normal |  | Present |  | Present |  |
| Day 9 |  |  | Small |  | Small |  |  |  | 35.4 |  | 10.4 |  |  |  | Present |  | Present |  |
| Day 10 | between 3-4 | | | | | | between 32-36 | | | | | | Normal | | | | | |
| Day 11 | 3.2 |  | 2.0 |  | 2.1 |  | 32 |  | 32 |  | 24.6 |  | Normal | | | | | |
| Day 12 | 3.4 |  | 2.0 |  | 2.1 |  | 32.7 |  | 27.3 |  | 15.7 |  | Normal | | | | | |
| Day 13 | 3.8 |  | 2.8 |  | 2.5 |  | 32.2 |  | 18.7 |  | 12.8 |  | Normal | | | | | |
| Day 14 | 3.8 |  | 3.2 |  | 2.6 |  | 30 |  | 29 |  | 31 |  | Normal | | | | | |
| Day 15 | 3.8 |  | 2.3 |  | 2.2 |  | 57.8 |  | 26.9 |  | 24.2 |  | Normal | | | | | |
| Day 16 |  |  |  |  |  |  | 32.7 |  | 16.6 |  | 20 |  | Normal | | | | | |
| Day 17 | 3.2 | 3.1 | 2.0 | 1.8 | 2.2 | 2.3 | 30-40 | | 35 | 20 | 26 | 25 | Normal | | | | | |
| Day 18 | 3.2 |  | 2.1 | 1.9 | 2.0 | 2.2 | 45.7 |  | 50 | 34 | 44 | 39.6 | Normal | | | | | |
| Day 19 | 4.6 |  | 2.2 | 1.9 | 2.4 | 2.4 | 35 |  | 51 | 30 | 35 | 35 | Normal | | | | | |
| Day 20 | 4.8 |  | 2.5 | 2.4 | 2.4 | 2.4 | 34 |  | 56 | 13 | 28 | 33 | Normal | | | | | |
| Day 21 | 5.1 |  | 3.0 | 3.0 | 2.8 | 2.7 | 30.5 |  | 33 | 35 | 18 | 22 | Normal | | | | | |
| Day 22 | 4.4 |  | 2.3 | 2.0 | 2.6 | 2.7 | 27.3 |  | 30 | 17 | 33.1 | 28.2 | Normal | | | | | |
| Day 23 | 4.7 | 3.6 | 2.0 | 1.7 | 2.0 | 2.3 | 47 | 45.2 | 30 | 28 | 34.9 | 29.6 | Normal | | | | | |
