# Supplementary material for: Vascular insufficiency in the extremities following jellyfish-sting envenomation in Malaysia
Source: Toxicon X. 2026 Jan 21;29:100239. doi: 10.1016/j.toxcx.2025.100239 (PMC12870873; doi:10.1016/j.toxcx.2025.100239)
Supplement: Multimedia component 2 [file mmc2.docx]

S2 Table : Case 4 serial Doppler ultrasound findings. Arteries name abbreviations: Right brachial artery (RBA), Right radial artery (RRA), Right ulnar artery (RUA), Dorsalis pedis artery (DPA), posterior tibial artery (PTA)

| Day of incident | Caliber (cm) | | | | | | | Colour doppler / PSV (cm/sec) | | |
| --- | --- | --- | --- | --- | --- | --- | --- | --- | --- | --- |
|  | RBA | RRA | RUA | Right DPA | Right PTA | Left DPA | Left PTA | RBA | RRA | RUA |
| Day 3 |  |  |  |  |  |  |  | Triphasic (100.2 cm/sec) | Monophasic (23cm/sec) | Biphasic (51.7cm/sec) |
| Day 4 |  | 0.23cm  (mid portion) | 0.22cm  (mid portion) | small | small | small | 0.22cm |  |  |  |
| Day 5 | Vasospasm of long segment non-opacification of RRA and RUA. | | |  |  |  |  |  |  |  |
| Day 6 |  | 0.23cm  (mid portion) | 0.22cm  (mid portion) | 0.16cm | 0.15cm | 0.16cm | 0.22cm |  |  |  |
| Day 7 |  | 0.15 x  0.23cm  (wrist) | 0.22 x  0.25cm  (wrist) | 0.19 x  0.32cm | 0.27 x 0.28cm | 0.15 x 0.22cm | 0.26 x 0.34cm |  |  |  |
| Day 11 |  | 0.19cm | 0.16cm | 0.2cm | 0.21cm | 0.15cm | 0.21cm |  |  |  |
